# Role of pheromone recognition systems in creating new species of fission yeast

**DOI:** 10.15698/mic2019.04.675

**Published:** 2019-03-11

**Authors:** Taisuke Seike, Chikashi Shimoda

**Affiliations:** 1 Genetics Strains Research Center, National Institute of Genetics, Shizuoka, Japan.; 2 Graduate School of Science, Osaka City University, Osaka, Japan.

**Keywords:** pheromone, fission yeast, G-protein coupled receptor, reproductive isolation, speciation, diversification, coevolution

## Abstract

Many species, from mammals to microorganisms, release sex pheromones to attract a potential partner of the opposite sex. The combination of a pheromone and its corresponding receptor determines the species-specific ability of males and females to recognize each other, and therefore causes reproductive isolation. This barrier, which has arisen to restrict gene flow between mating pairs, might facilitate reproductive isolation leading to incipient speciation, but how do new combinations of pheromone and receptor evolve? Our recent study demonstrated an “asymmetric” pheromone recognition system in the fission yeast *Schizosaccharomyces pombe*: among the two pheromone/receptor pairs in this yeast, recognition between one pair is stringent, while that between the other pair is rather relaxed. We speculate that the asymmetric properties of these pheromone recognition systems are beneficial for gradual evolution resulting in reproductive isolation in yeasts.

The two mating-type cells (**P**lus and **M**inus) of *S. pombe* secrete two different peptide pheromones (P-factor and M-factor), which are recognized by their cognate receptor on the surface of cells of the opposite type. The mating reaction between P- and M-cells requires both recognition of P-factor by its receptor (Mam2) and recognition of M-factor by its receptor (Map3). We previously created a novel M-factor/Map3 mutant pair that is compatible with one other, but not with their original wild-type counterparts, by genetically altering the primary structure of both M-factor and Map3. The resulting yeast expressing the novel pair was reproductively isolated from the wild-type population. This experimental result supports the idea that changes in pheromone/receptor recognition may be a potential mechanism underlying reproductive isolation in nature. A loss-of-function mutation in a pheromone gene would prevent appropriate recognition of the pheromone with its receptor, but how would a pheromone diversify and maintain successful mating in nature? To answer this question, we explored the genes encoding the two pheromones and their receptors in 150 strains of *S. pombe* that originated from different geographic areas of more than 22 countries. Sequencing analysis clearly demonstrated that there is no variation in the amino acid sequences of one pair, M-factor and Map3, while the other pair, P-factor and Mam2, show substantial diversity. Interestingly, such asymmetric diversification of the two pheromones is also seen in two fission yeast laboratory strains *S. cryophilus* and *S. octosporus*, implying that communication by M-factor plays an important role in defining the species, rather than signaling by P-factor.

Wild *S. pombe* strains have three M-factor-encoding genes and four to eight copies of P-factor-encoding sequence in a single gene. Such genetic redundancy might ensure mating even if one copy of the pheromone gene is altered. Our previous work revealed that comprehensive substitutions could be made in the N-terminal half of M-factor without much effect on biological activity, and even mutants with a deletion of several residues at the N-terminus of M-factor retained substantial activity. In addition, the deletion of any one or two of the three M-factor genes led to no marked defects. Notably, however, the amino acid sequences of M-factor (and Map3) are completely conserved in the wild strains that we investigated. Furthermore, *S. cryophilus* and *S. octosporus* have, respectively, five and six genes for M-factor, each encoding an identical amino acid sequence of mature M-factor peptide. By contrast, we found non-synonymous variants of P-factor peptides, with wild *S. pombe* strains producing a mixture of three distinct mature P-factor peptides, and the closely related *S. cryophilus* and *S. octosporus* producing, respectively, three and four distinct mature peptides as a mixture. In principle, both pheromones have the ability to evolve, but somehow M-factor has been conserved. Below, we propose a hypothesis for asymmetric diversification of the two pheromones based on our following experimental observations.

First, *S. octosporus* M-factor (So-M-factor) stimulated no mating response from wild-type *S. pombe*, but introduction of *S. octosporus* P-factor (So-P-factor) could restore physiological mating to approximately 50% of wild-type efficiency in a laboratory *S. pombe* strain lacking its own P-factor gene. Similarly, introduction of Sp-P-factor could partially restore mating in a laboratory *S. octosporus* strain lacking its own P-factor gene. Indeed, most of the P-factor variants were found to have physiological effects between *S. pombe* and *S. octosporus*, whereas M-factor was dysfunctional in other species. In other words, M-factor operates only within the same species, whereas P-factor operates beyond species (**Fig. 1**). These results suggest that two pheromone receptors might have distinct specificities for their corresponding pheromone. The two pheromone receptors, Mam2 and Map3, are so-called “class D” G-protein coupled receptors (GPCRs), and show sequence homology to, respectively, the Ste2 α-factor receptor and Ste3 a-factor receptor of the budding yeast *Saccharomyces cerevisiae*. Greig and colleagues recently reported that a-factor variants show all-or-none effects on mating ability, whereas α-factor variants exhibit more gradual effects in *S. cerevisiae*. We speculate that distinct differences in the ligand specificity of the two GPCRs, such as stringent binding-pocket requirements, facilitate the asymmetric diversification of pheromones in yeasts.

**Figure 1:**
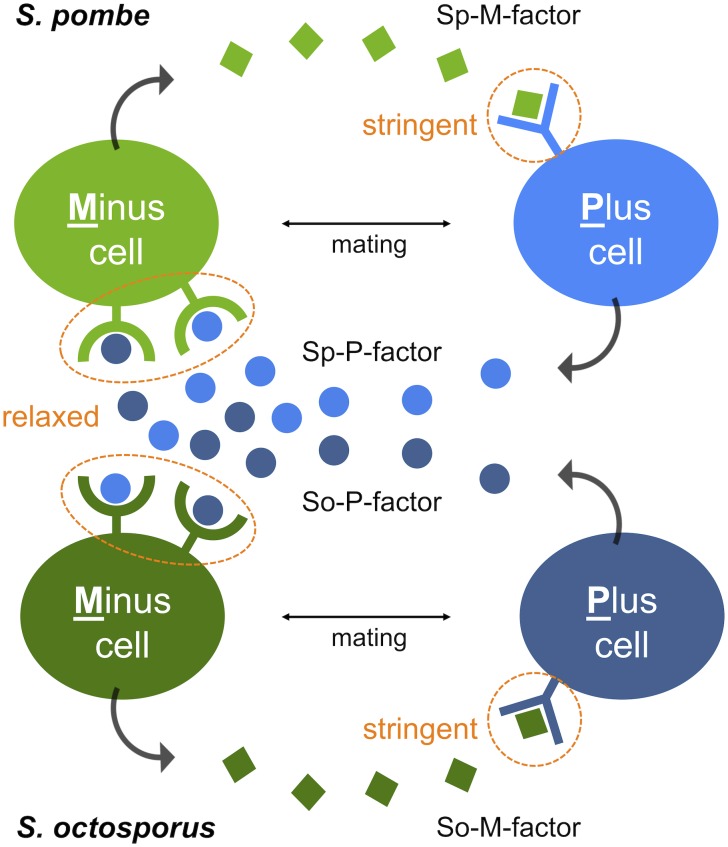
FIGURE 1: M-factor function is stringent, whereas P-factor function is more relaxed, extending beyond species. M-factor and P-factor, secreted by **M**inus and **P**lus cells, respectively, are recognized by their corresponding receptor on the surface of cells of the opposite mating type. Whereas the specificity of M-factor recognition is extremely stringent, that of P-factor recognition is more relaxed, allowing cross-reactions to occur even between *S. pombe* and *S. octosporus*. P-factor is interchangeable between the two species, but not M-factor. Sp, *S. pombe*; So, *S. octosporus*.

Second, the two pheromone peptides of *S. pombe* differ considerably in chemical structure: P-factor is a simple peptide, whereas M-factor is a farnesylated (lipid) peptide that is highly conserved in ascomycetes. In addition, P-factor is degraded by a specific protease (Sxa2), whereas M-factor is not. The existence of such a protease might facilitate one-sided diversification of the P-factor and Mam2 pair, because both Mam2 and Sxa2 compete in binding to the same regions (residues) of P-factor (**Fig. 2**).

**Figure 2:**
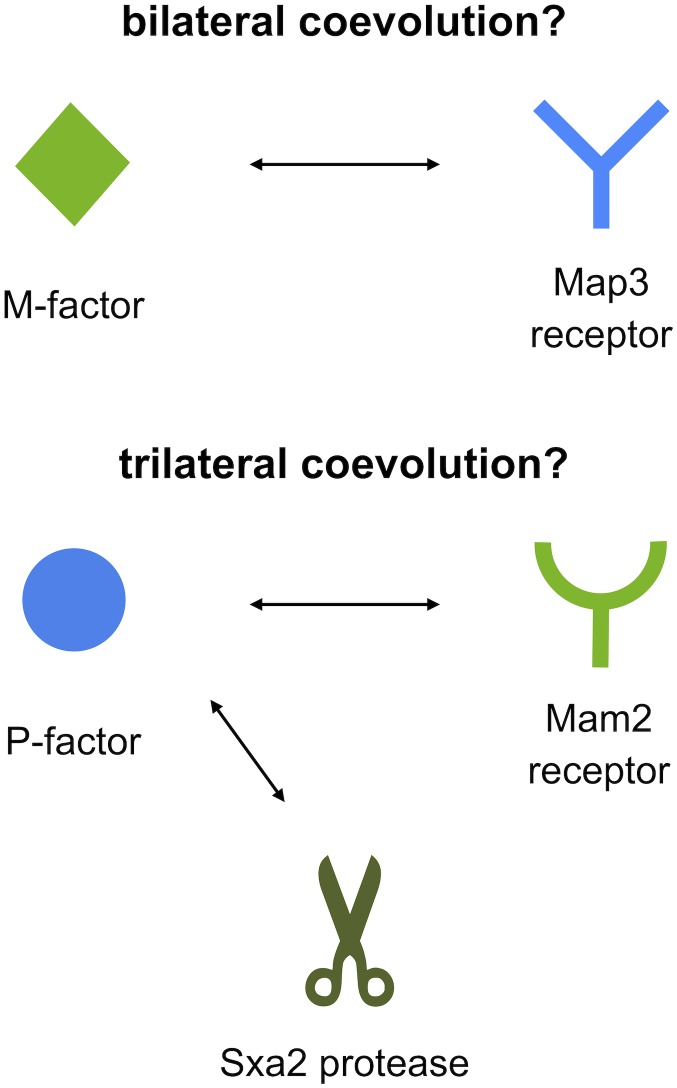
FIGURE 2: Model of the asymmetric diversification of two mating pheromones in terms of specific proteases. M-factor is recognized by the Map3 receptor, leading to ‘bilateral' coevolution. By contrast, P-factor is recognized by both the Mam2 receptor and the Sxa2 protease, perhaps leading to ‘trilateral' coevolution coupled with P-factor divergence, thereby facilitating more rapid diversification of P-factor relative to M-factor.

At present, the exact reason why *S. pombe* possesses an asymmetric pheromone recognition system remains unclear, but it might allow flexible adaptation to genetic changes in pheromones, while maintaining stringent recognition by the lipid peptide, perhaps leading to reproductive isolation slowly but surely. Our findings contribute new insight into the evolutionary mechanisms underlying the diversification of pheromones. Organisms in general might have similar systems for creating new versions of pheromones, allowing them to persist long enough in a population to evolve adaptations of receptors. Before a mutant is completely lost, a second suppressor mutation may occur to recover the first defect. Thus, the coevolution of pheromone/receptor pairs might proceed step by step.

